# Arthroscopic Repair of Posterior Root Tear of the Lateral Meniscus Using the Anterior Meniscofemoral Ligament

**DOI:** 10.1002/atn2.70110

**Published:** 2026-07-21

**Authors:** Robert Śmigielski, Jan Mateńko, Daniel Kopko

**Affiliations:** ^1^ Life Institute Warsaw Poland

## Abstract

Posterolateral meniscus root tears are frequently associated with anterior cruciate ligament injuries and contribute to altered knee biomechanics, increased joint loading, and potential graft failure. Despite various described repair techniques, the use of the anterior meniscofemoral ligament (aMFL, ligament of Humphrey) for repair is not widely popular, a structure that remains intact in most posterolateral meniscus root tears cases and shows strong anatomical and biomechanical integration with the posterior horn of the lateral meniscus. This article describes an arthroscopic technique utilizing the aMFL for the anatomical reconstruction and stabilization of lateral meniscus posterior root tears. This method aims to improve fixation strength and biological healing by leveraging an existing ligament with a favorable insertion trajectory and structural continuity with the meniscal root. The aMFL is arthroscopically dissected, sutured at its femoral attachment and then detached above the suture. A tibial tunnel is placed in the area of the remnants of the posterior root; then, the aMFL is shuttled through the tunnel and fixated within it using an interference screw or in the hybrid form with extracortical fixation.

## Graphical abstract

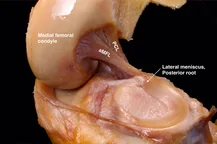

**VIDEO 1:** The video shows the following steps: identification of the posterior root tear of the lateral meniscus; view after removal of the damaged anterior cruciate ligament and dissection of the anterior meniscofemoral ligament (aMFL); debridement of the posterior root tear and preparation of the site for tibial tunnel creation; exposure of the aMFL and visualization of its connection with the lateral meniscus; placement of sutures near the femoral attachment of the aMFL; sectioning of the aMFL proximal to the sutures; preparation and drilling of the tibial tunnel; passage of the guiding suture through the drilled tunnel; retrieval of the guiding suture through the arthroscopic portal and its connection to the aMFL suture; pulling of the aMFL into the tibial tunnel; and final view showing the secured posterior root of the lateral meniscus. Video content can be viewed at https://doi.org/10.1002/atn2.70110.

This technical note describes an arthroscopic technique for repairing posterior root tears of the lateral meniscus using an existing anatomical structure, the anterior meniscofemoral ligament (aMFL, ligament of Humphrey). By utilizing this native ligament in the repair process, the technique aims to enhance healing potential and improve fixation strength by leveraging the biomechanical support of an already present structure. This method may offer a simplified and biologically favorable alternative to conventional repair strategies.

Posterolateral meniscus root (PLMR) tears are mainly traumatic injuries that frequently occur with anterior cruciate ligament (ACL) tears.^1^ The PLMR plays a crucial role in stabilizing the knee by limiting anterior tibial translation during low flexion angles and controlling internal rotation at higher flexion angles. Deficiency of this structure may lead to increased knee instability, contributing to greater functional limitations and potentially elevating stress on ACL reconstruction grafts.^2^ A posterior horn detachment of the lateral meniscus has been shown to significantly increase peak contact pressure in the lateral compartment, indicating its essential role in load distribution within the knee joint.^3^ The risk factors associated with lateral meniscal posterior root tears remain incompletely understood. Previous studies have indicated that approximately 87% of lateral meniscal injuries are related to sports activities, with Beldame et al. reporting that 70% of such injuries occur during pivot‐contact sports.^4^, ^5^ The incidence of posterolateral meniscus root tears was 6.6% in a large series of patients undergoing ACL reconstruction. Participation in contact sports and the presence of a concomitant medial meniscal tear were shown to be important independent risk factors. Their presence should raise the index of suspicion of this injury pattern.^6^

Surgical repair of the PLMR is indicated to stabilize the knee and prevent the progression of osteoarthritis. The literature describes a variety of surgical techniques for treating posterior root tears of the lateral meniscus. Notably, upon analysis, the use of the whole aMFL in repair is not widely employed, which is the focus of the current technical note.^7^, ^8^, ^9^, ^10^, ^11^, ^12^, ^13^ Zaffagnini et al. showed a surgical technique utilizing the medial fibers of the posterior cruciate ligament (PCL) as an anchor can mimic the function of the meniscofemoral ligament (MFL) to restore posterior root stability. Although the MFL is not directly sutured, its biomechanical role is functionally replicated.^14^ Knapik et al. describe that intact MFLs may reduce the necessity for lateral meniscus root repair in certain ACL‐injured knees and advocate for individualized surgical decision‐making based on the presence of the MFL.^15^

Anatomical fixation of the PLMR using standard suturing methods is often challenging due to the tear's location. Therefore, utilizing the existing and typically intact aMFL appears to be a technically more efficient approach and may also offer improved biological outcomes in the healing process. As Ahsan et al. say, even in the presence of a lateral meniscus posterior root (PLMR) tear, the aMFL is commonly found to remain intact during intraoperative assessment.^16^ This pattern has also been consistently observed in our clinical practice. Pekala et al. in their study, reported the highest prevalence of the aMFL in arthroscopic studies (82.3%), followed by cadaveric dissections (60.0%) and magnetic resonance imaging assessments (35.8%).^17^ The overall pooled prevalence was 55.5%. The presence of an intact aMFL in cases of meniscal injury provides a rationale for considering the application of the technique described in this technical note.

## SURGICAL TECHNIQUE

### Anatomy and Function of aMFL

**FIGURE 1:**
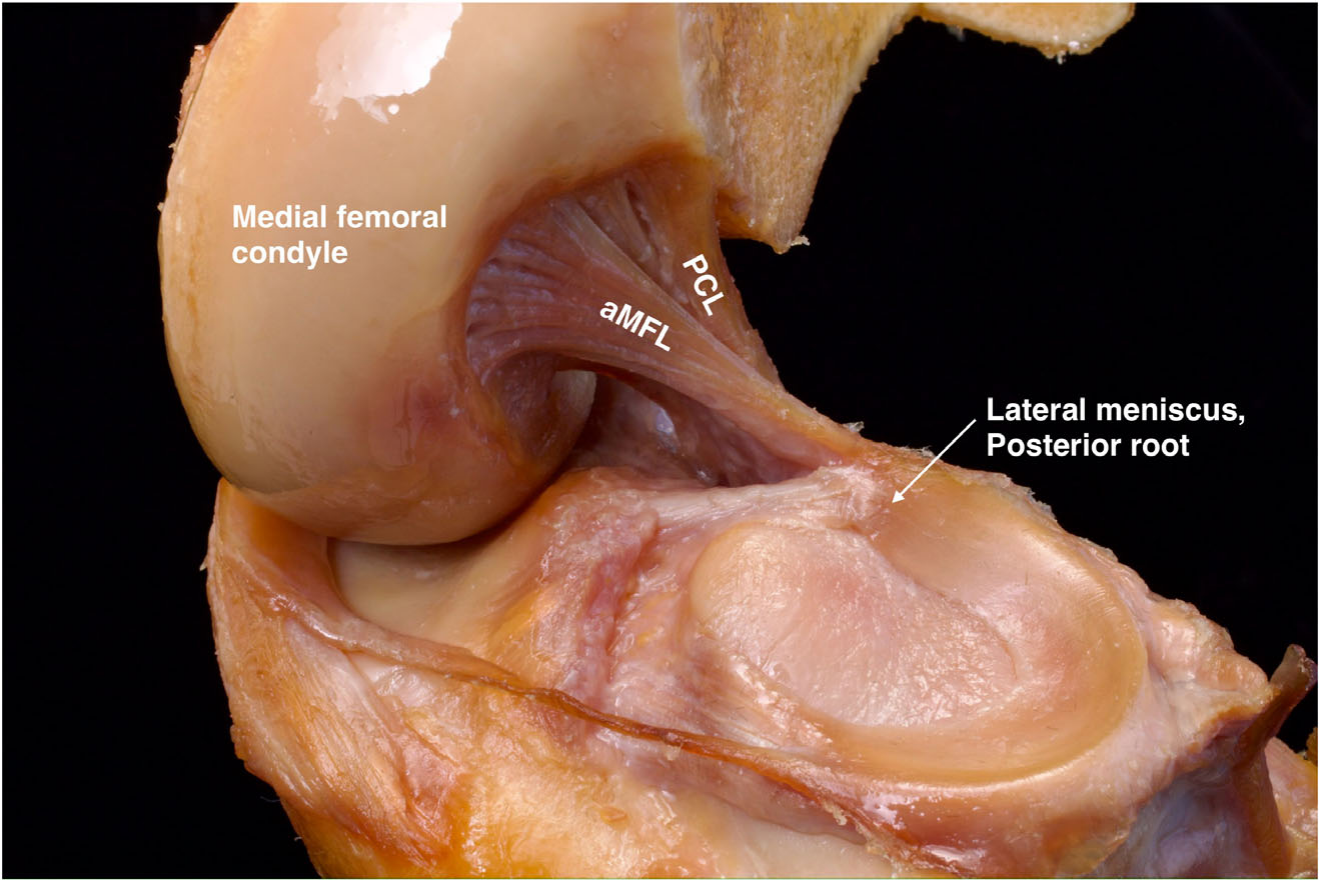
Anatomy of left aMFL. The course of the ligament is visible, including its meniscal and femoral attachments. Specimen preparation, photograph and anatomical labeling by the authors. (aMFL, anterior meniscofemoral ligament; PCL, posterior cruciate ligament.)

**FIGURE 2:**
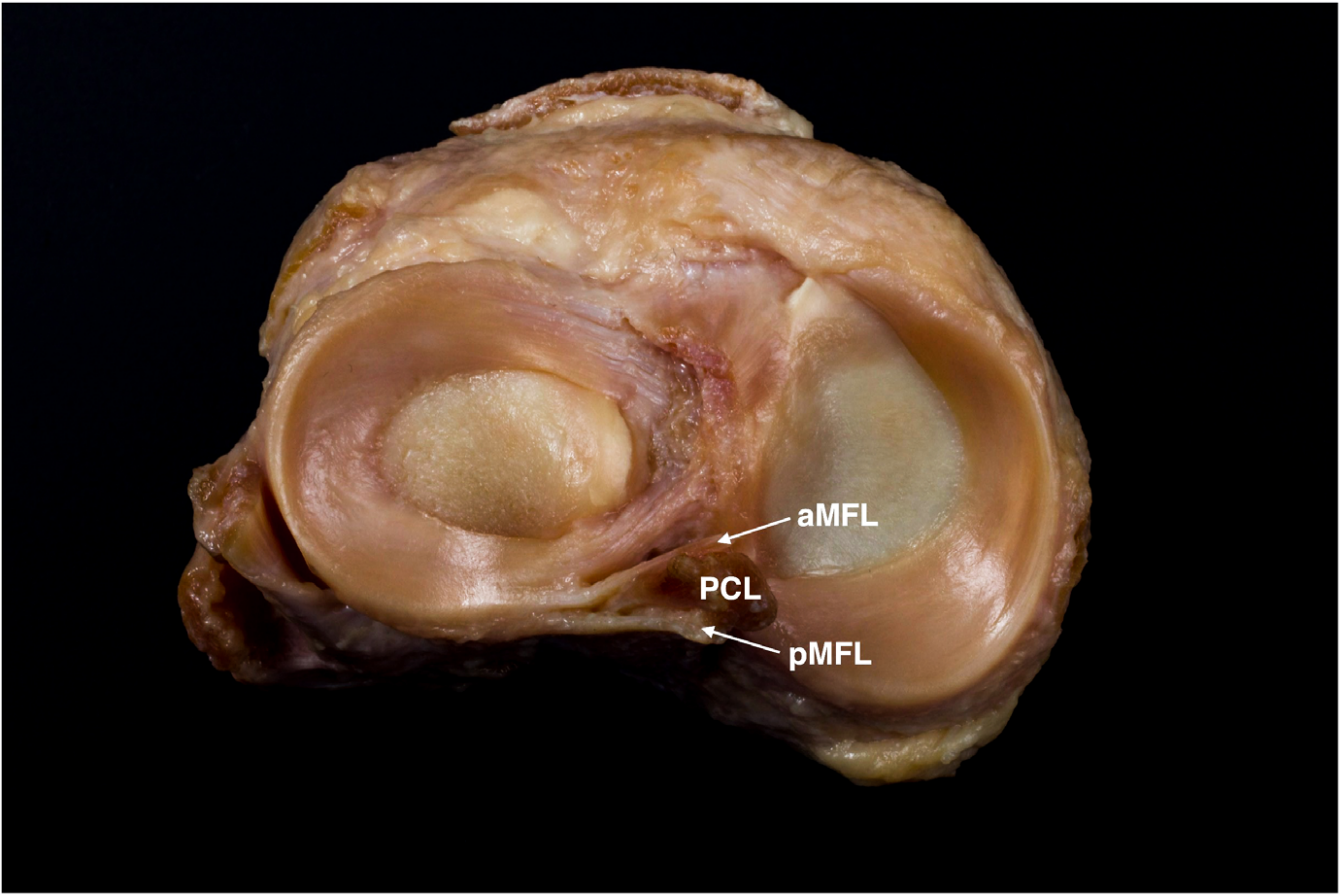
aMFL and pMFL. Specimen preparation, photograph and anatomical labeling by the authors (Left knee). (aMFL, anterior meniscofemoral ligament; PCL, posterior cruciate ligament; pMFL, posterior meniscofemoral ligament.)

**FIGURE 3:**
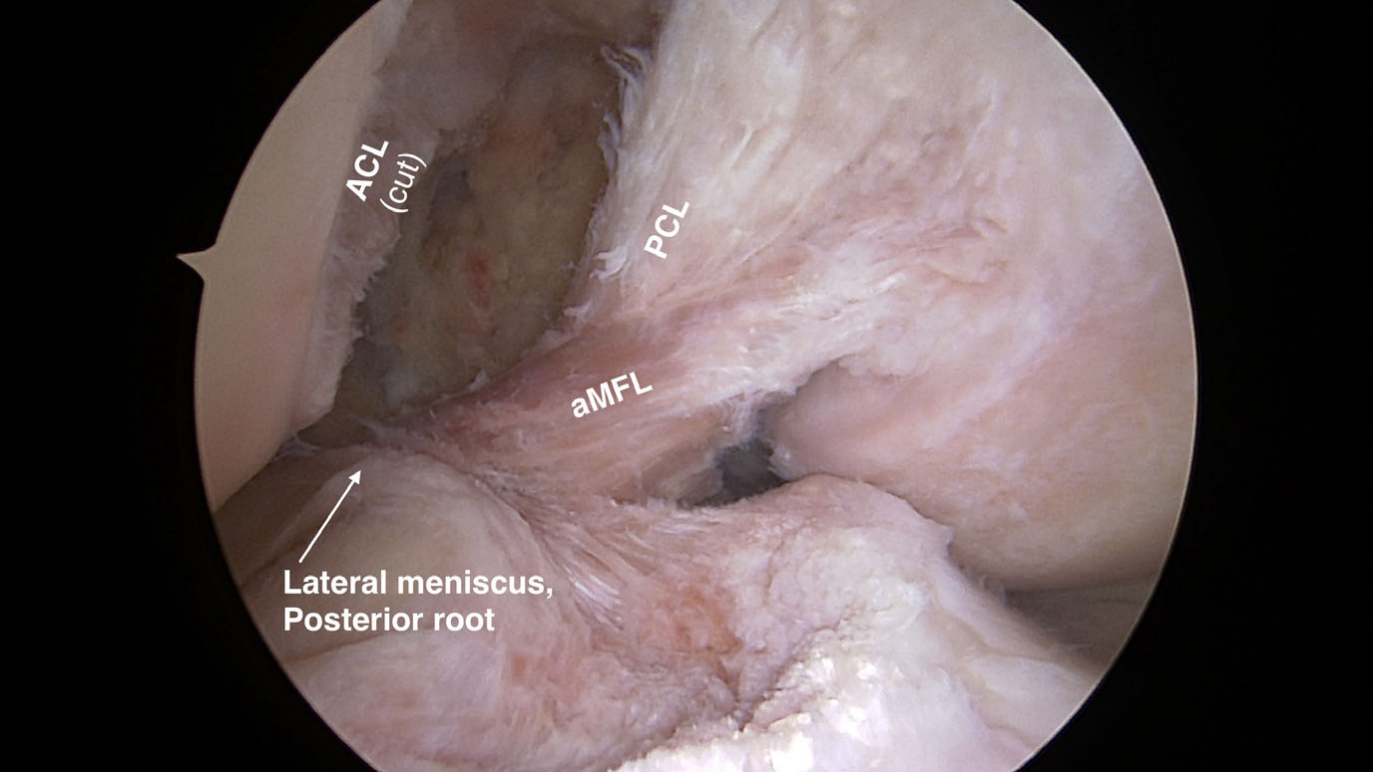
Arthroscopic view of an anatomical knee joint specimen. Specimen preparation, photograph, and anatomical labeling by the authors (Right knee). (ACL, anterior cruciate ligament; aMFL, anterior meniscofemoral ligament; PCL, posterior cruciate ligament.)

The posterior horn of the lateral meniscus has a dual insertion: the anterior portion attaches to the tibial intercondylar eminence, while the posterior portion, in most cases, inserts into the femur via the MFLs, primarily the aMFL (Figures 1, 2, 3, Video 1). This anatomical configuration enables the aMFL to exert a medial and slightly anterior pull on the posterior horn, thereby enhancing femoromeniscal‐tibial congruence. The 2 MFLs connect the posterior horn of the lateral meniscus to the medial aspect of the femoral condyle as distinct anatomical structures, each with separate femoral and meniscal attachment sites.^18^ The aMFL traverses obliquely across the distal aspect of the PCL in the flexed knee. It originates from the femur distal to the PCL, making it superficially located when the knee is flexed. The ligament lies adjacent to the articular cartilage, approximately at the 10 o'clock position in the left knee, and its fibers interweave with those of the PCL near their femoral insertions. The distal attachment of the aMFL is located on the posterior horn of the lateral meniscus. Although this insertion is challenging to visualize in the intact knee, it can be clearly shown when the ACL is excised, allowing anterior subluxation of the tibial plateau.^19^

There is an anatomical relationship between the posterior root of the lateral meniscus and the distal attachment of the aMFL, in which the aMFL functions as a structural extension of the posterior meniscal root. This anatomical continuity forms the basis for the described surgical technique, in which the aMFL is pulled into a tibial bone tunnel to achieve complete and stable fixation of the root. The biomechanical role of the aMFL is most prominent during knee flexion, when it exerts an anteromedial pull on the posterior horn of the lateral meniscus, thereby enhancing stabilization of the lateral meniscocondylar compartment. This protective mechanism, combined with the greater mobility of the lateral meniscus, may help explain the lower incidence of lateral meniscal tears compared with the medial meniscus, which lacks a similar structural support.^17^

It has been shown by Forkel et al. that the aMFL compensates for PLMR injury by maintaining intra‐articular pressure equilibrium; therefore, root repair is recommended if the aMFL is compromised.^20^ Ahsan et al. introduced the humphrey intact, root out lesion and showed that the aMFL assumes a stabilizing load‐bearing role when the posterior root of the lateral meniscus cannot be anatomically repaired.^16^ Ohori et al., in an animal study, showed that the MFL shares load‐bearing function with the PLMR; thus, preserving the MFL during root repair is recommended to enhance root stability.^21^ Forkel et al., in their study, showed that transecting the MFL increases instability following an LMPR tear.^22^ They recommend concurrent root repair and emphasize the importance of assessing MFL integrity during surgical planning.

The analysis of the aforementioned studies clearly indicates that utilizing the integral and anatomical continuity between the posterior root of the lateral meniscus and the aMFL in surgical treatment offers a promising approach for effectively managing PLMR injuries.

**FIGURE 4:**
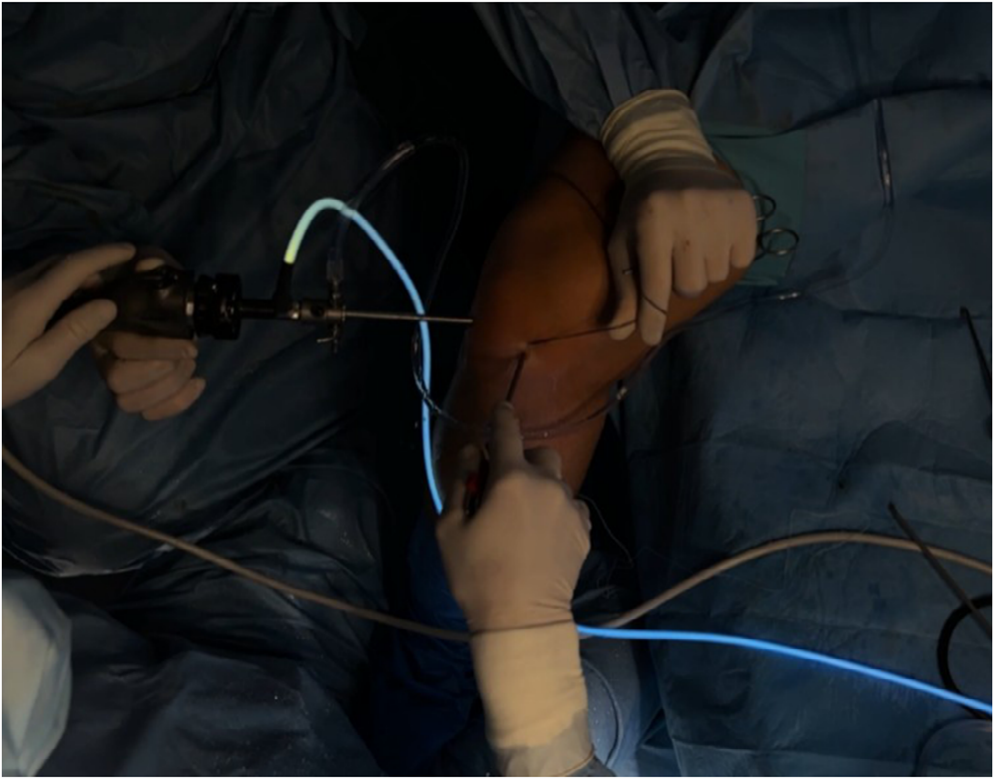
Intraoperative view of limb positioning during the surgical procedure (Right knee).

**FIGURE 5:**
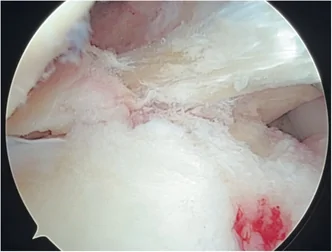
Visible tear of the posterior root of the lateral meniscus and the attachment of the aMFL (Right knee). (aMFL, anterior meniscofemoral ligament.)

**FIGURE 6:**
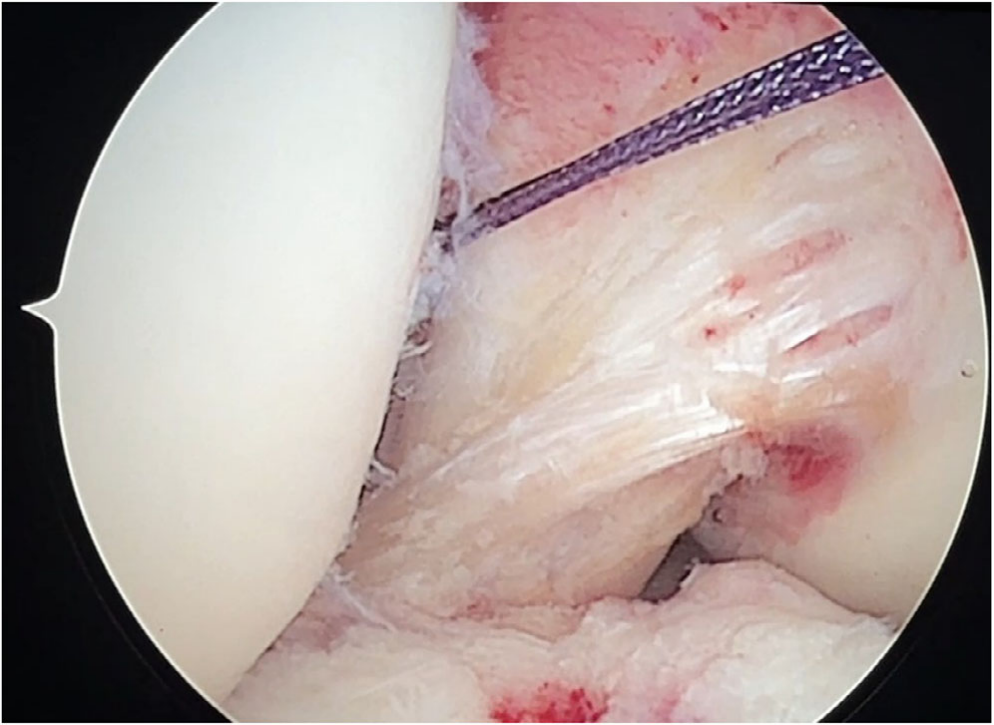
Femoral attachment of the aMFL (Right knee). (aMFL, anterior meniscofemoral ligament.)

**FIGURE 7:**
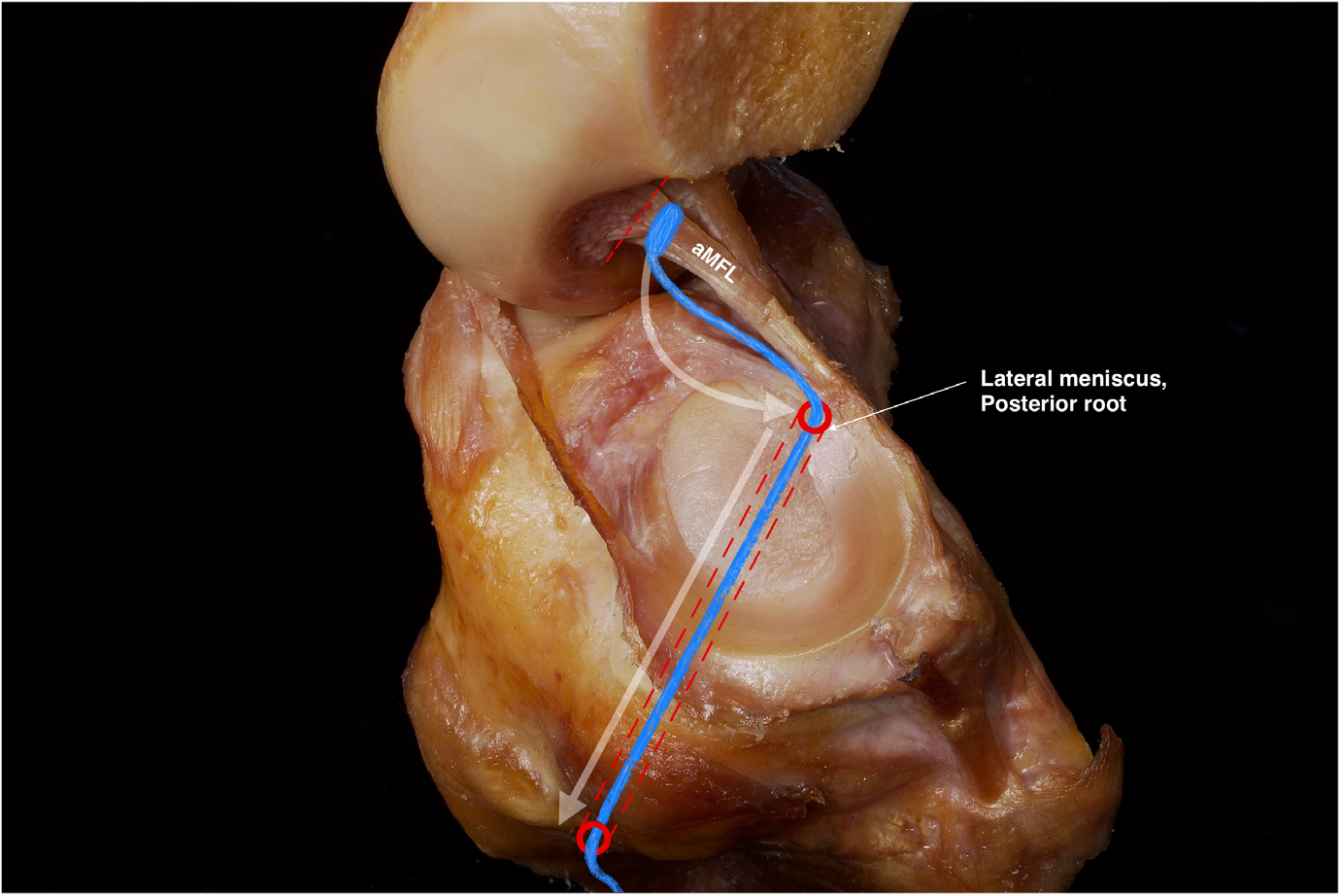
Schematic representation of the described technique. Anatomic preparation, photographs and captions prepared by the authors (Left knee). (aMFL, anterior meniscofemoral ligament.)

**FIGURE 8:**
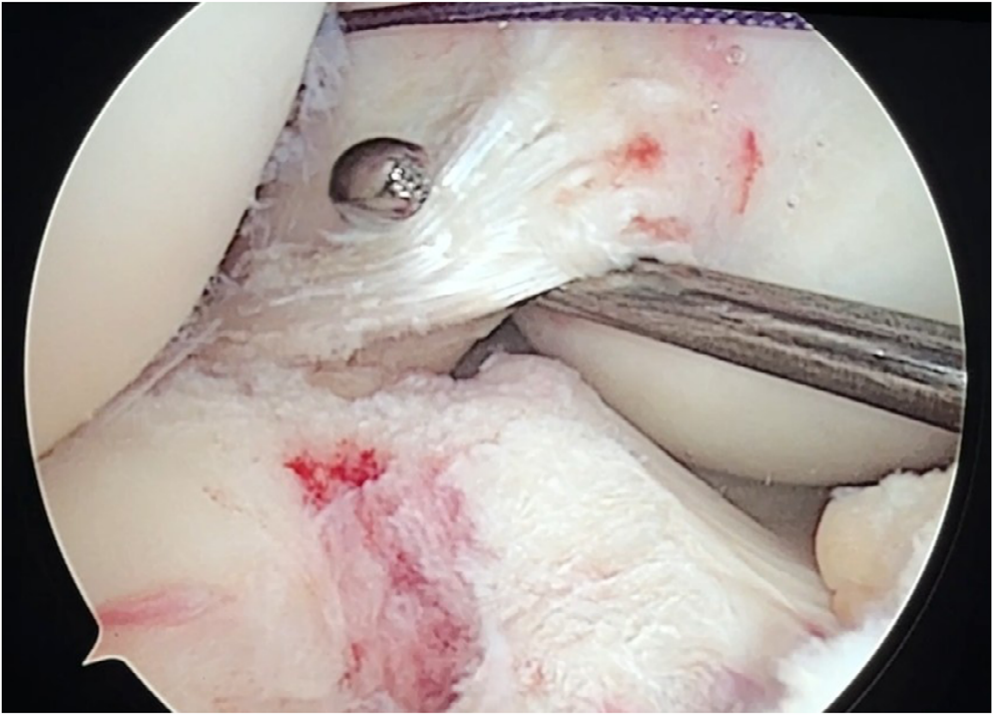
A view showing the exposed aMFL (Right knee). (aMFL, anterior meniscofemoral ligament.)

**FIGURE 9:**
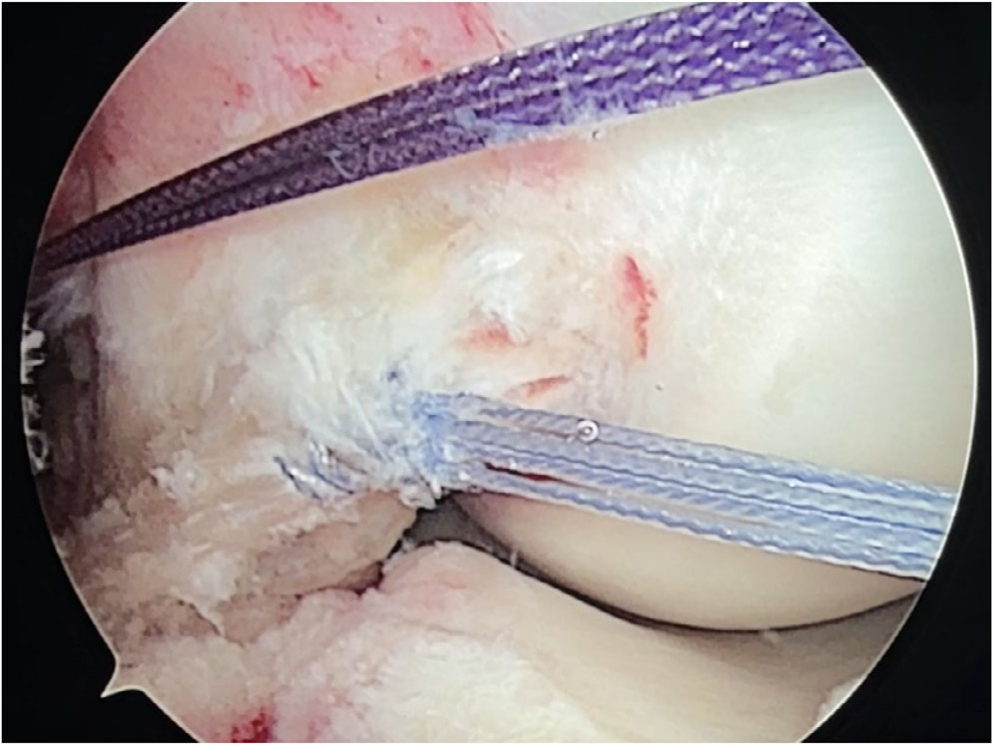
Placement of 2 sutures through the aMFL at its femoral attachment (Right knee). (aMFL, anterior meniscofemoral ligament.)

**FIGURE 10:**
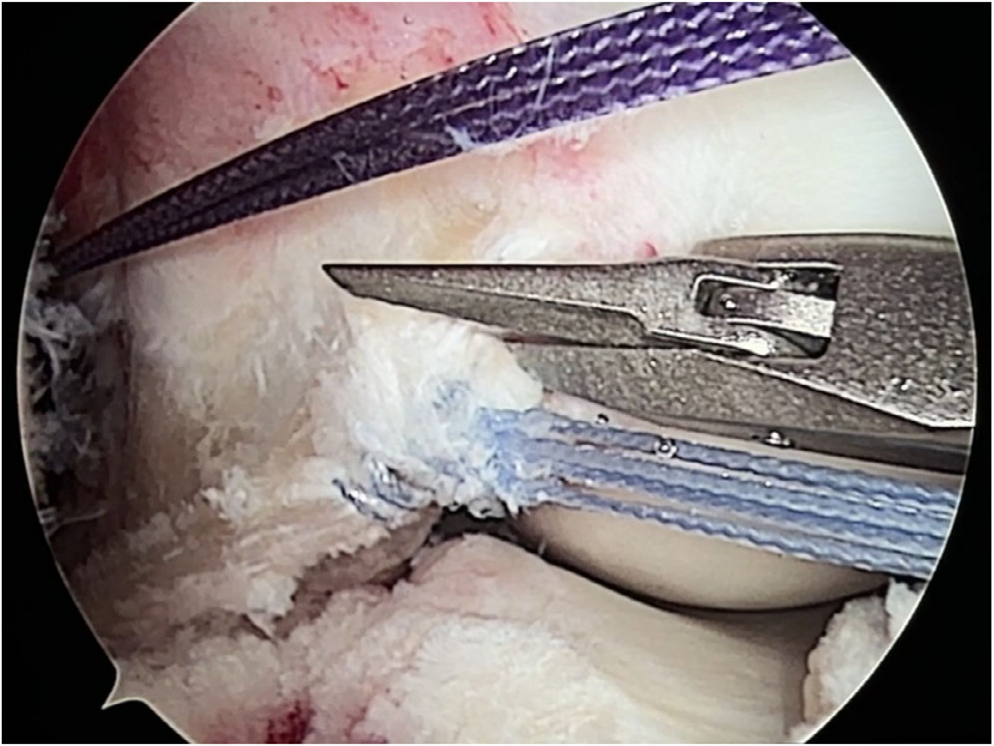
Transection of the aMFL from its femoral attachment proximal to the site of suture placement (Right knee). (aMFL, anterior meniscofemoral ligament.)

**FIGURE 11:**
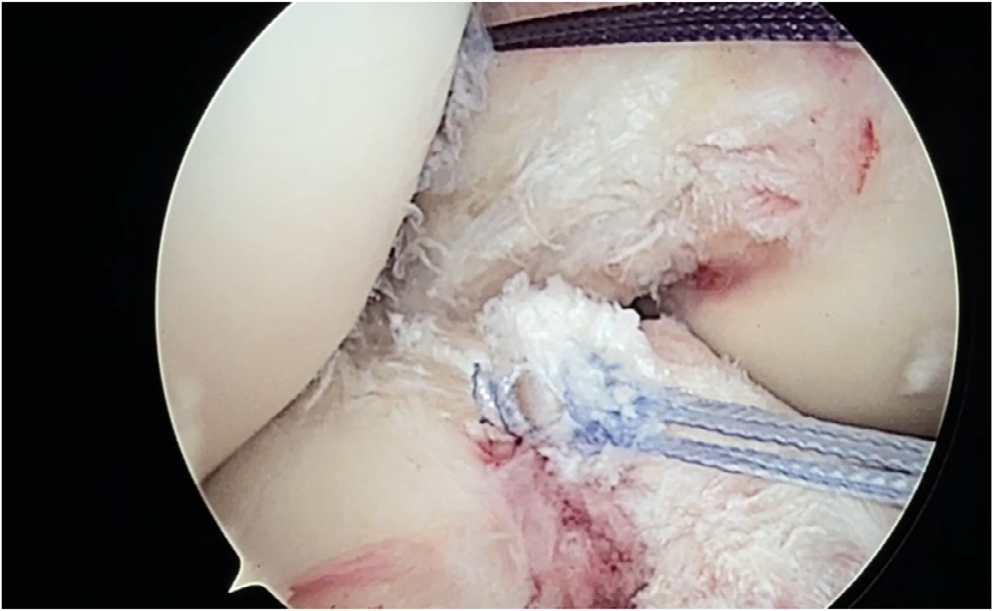
Released and prepared aMFL ready for passage into the tibial bone tunnel (Right knee).  (aMFL, anterior meniscofemoral ligament.)

**FIGURE 12:**
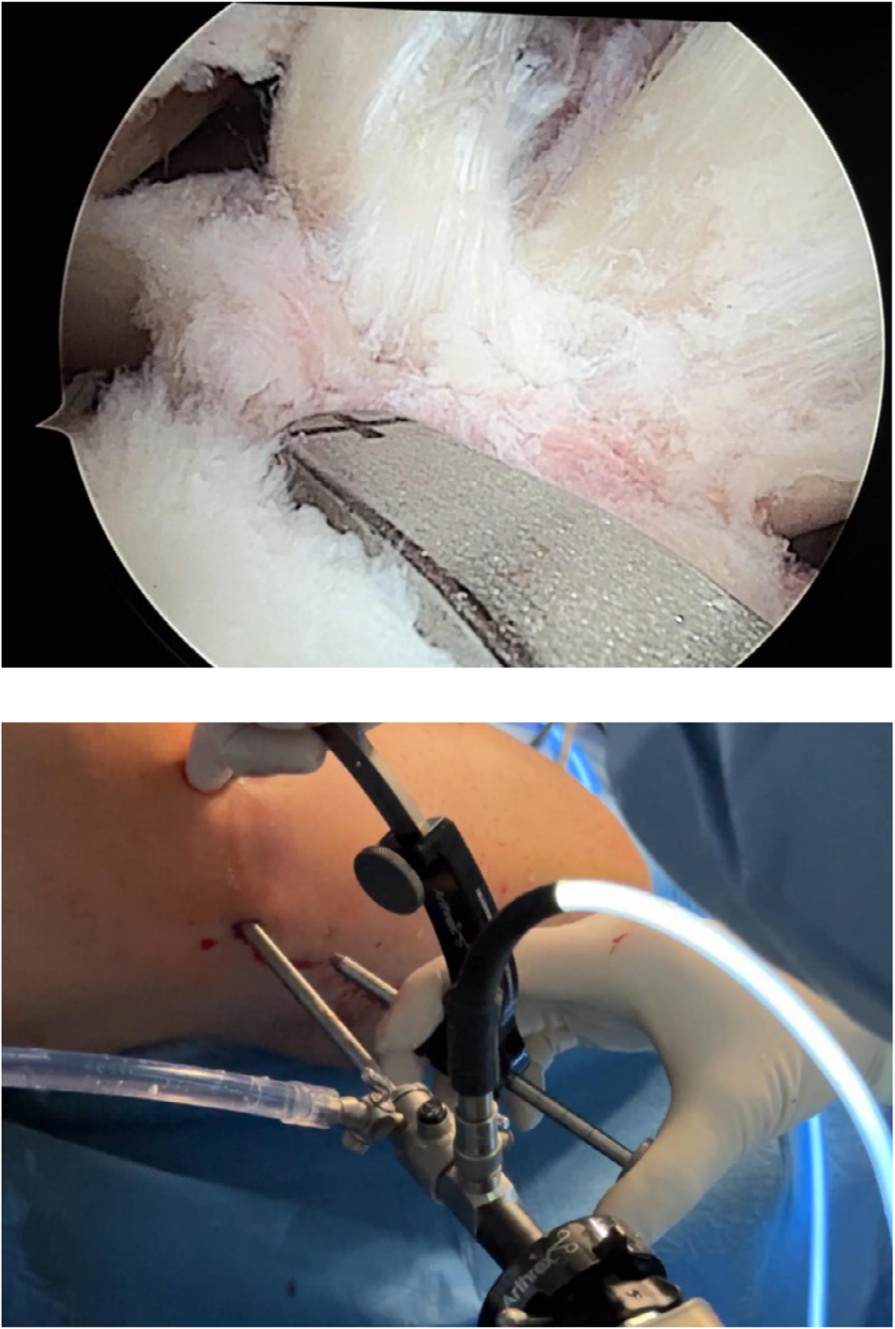
Positioning of the tibial tunnel entry point using a standard ACL aimer (Right knee). (ACL, anterior cruciate ligament.)

**FIGURE 13:**
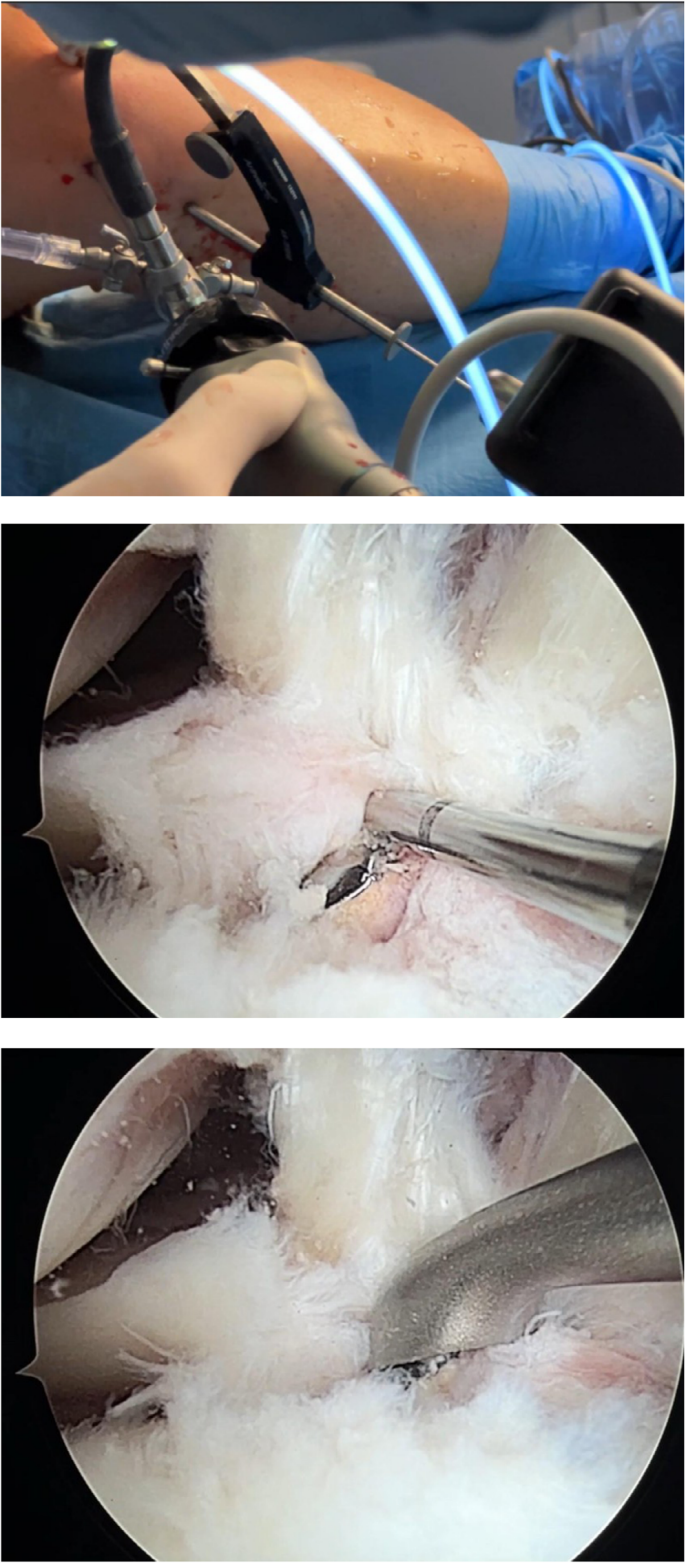
Drilling of the tibial tunnel (Right knee).

**FIGURE 14:**
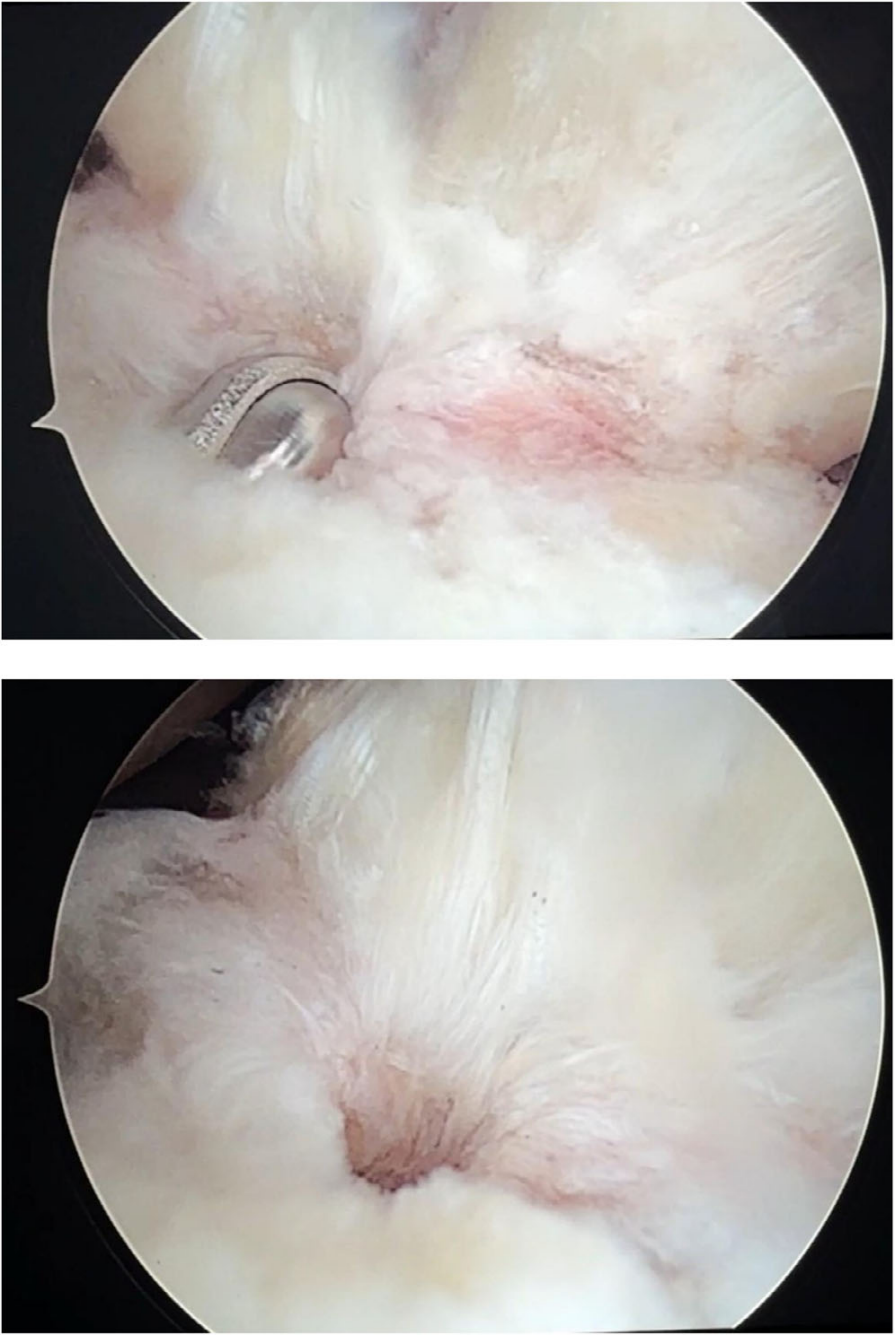
Debridement and preparation of the tibial tunnel using a shaver (Right knee).

**FIGURE 15:**
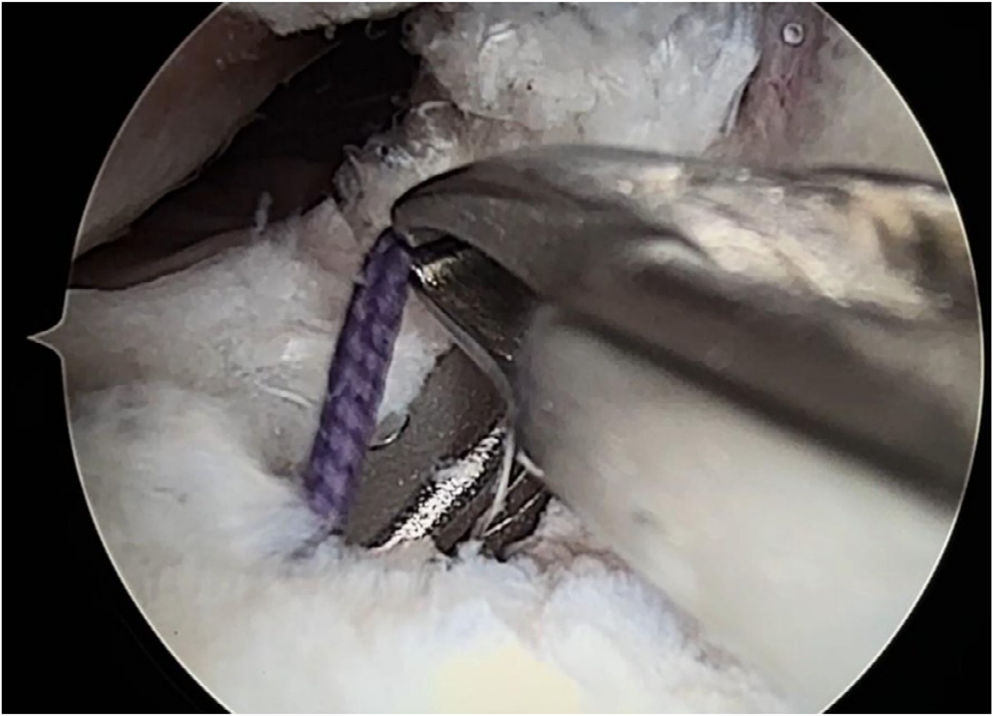
An additional guiding suture is introduced into the joint cavity through the distal aperture of the tibial tunnel and then exteriorized through one of the arthroscopic portals. This allows for extra‐articular knotting of the guiding suture with the suture previously attached to the aMFL (Right knee). (aMFL, anterior meniscofemoral ligament.)

**FIGURE 16:**
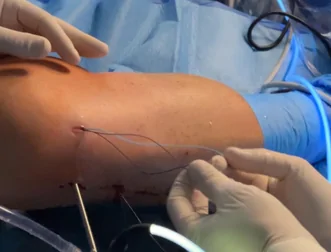
Temporary tying of the sutures outside the joint to create a guiding suture through the final tibial tunnel (Right knee).

**FIGURE 17:**
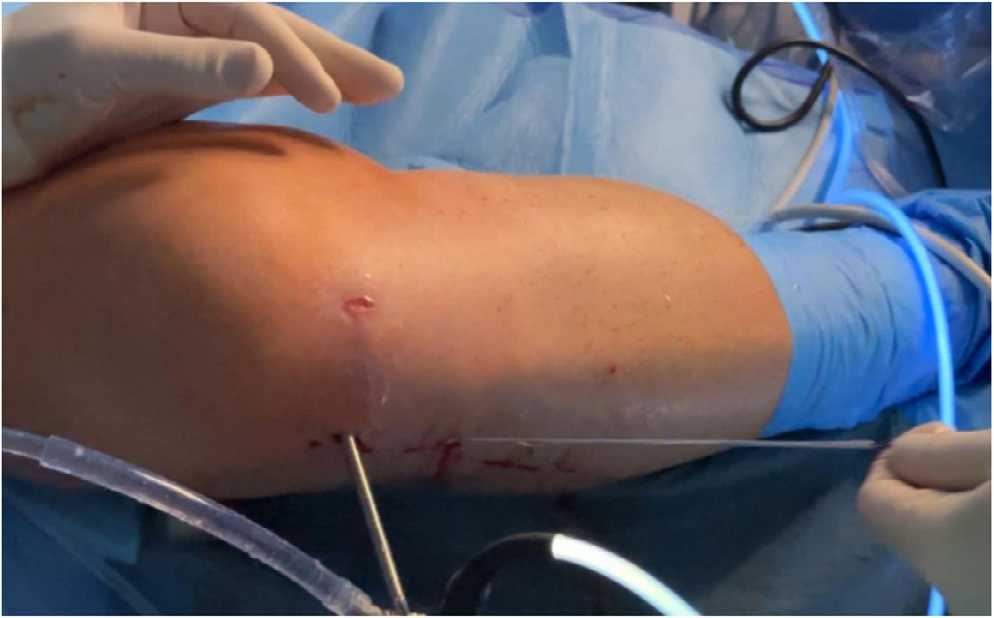
Pulling of the guiding suture through the tibial tunnel to draw the aMFL into its final position within the canal (Right knee). (aMFL, anterior meniscofemoral ligament.)

**FIGURE 18:**
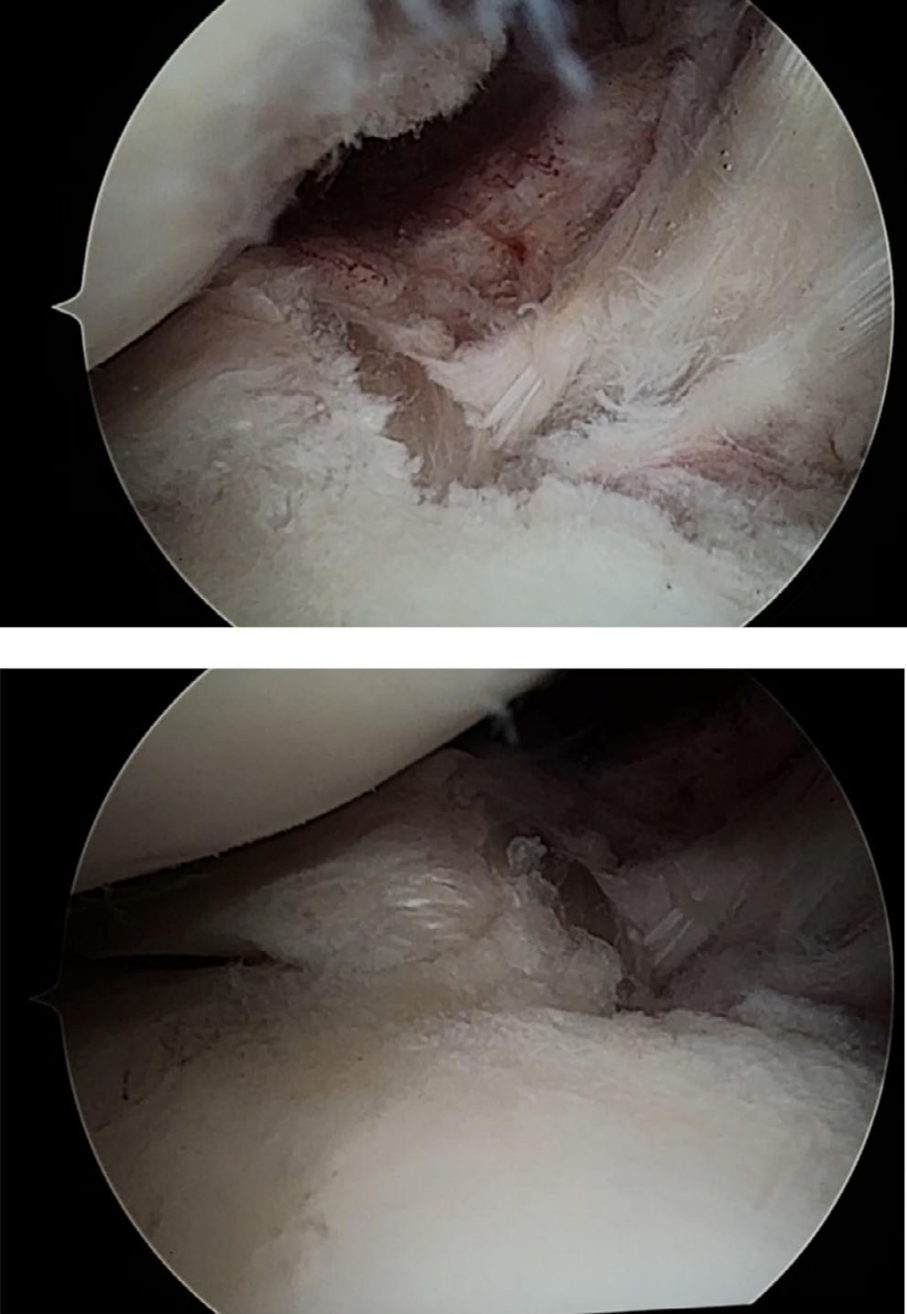
Visualized aMFL pulled into the tibial tunnel with concurrent fixation of the posterior root of the lateral meniscus (Right knee). (aMFL, anterior meniscofemoral ligament.)

**FIGURE 19:**
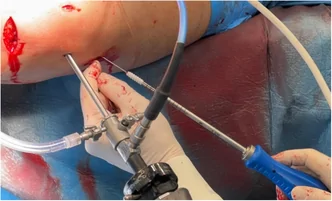
Fixation of the ligament within the bone tunnel using a hybrid technique combining both extracortical and interference screw fixation (Right knee).

In this case, fixation of the PLMR tear is performed concurrently with ACL reconstruction (Figures 4, 5, 6). The described technique involves arthroscopic dissection of the aMFL (Figures 7 and 8), followed by two passes of FiberWire sutures (Arthrex, Naples, FL), through the ligament at its femoral attachment site, using a Knee Scorpion Suture Passer (Arthrex, Naples, FL) (Figure 9). After suturing, the aMFL is detached from its femoral origin, using Arthrex Scissor (Figures 10 and 11). A tibial bone tunnel is then created, using a standard ACL aimer, with the intra‐articular entry point located on the posterolateral aspect of the tibial plateau, in the area of the remnants of the posterior root, and the extra‐articular exit positioned on the proximal tibia (Figures 12, 13, 14). Subsequently, an additional suture is introduced through the tibial tunnel and retrieved through one of the arthroscopic portals (Figure 15). The sutures previously placed at the femoral end of the aMFL are also passed out of the joint through the same arthroscopic portal. This allows the 2 sets of sutures to be tied together outside the joint (Figure 16), and by pulling on the suture exiting the tibial tunnel (Figure 17), the aMFL is drawn into the tunnel and fixated within it (Figures 18). Tibial tunnel fixation of the aMFL graft may vary depending on the surgeon's preference and can be performed using one of the following techniques: extracortical fixation, interference screw fixation, or a hybrid technique combining both extracortical and interference screw fixation. In our case, a hybrid fixation method was employed, which allowed for earlier postoperative mobilization and earlier weight‐bearing of the operated limb (Figure 19).

## Biomechanical Consequences of aMFL Resection for the Knee Joint

A fundamental question regarding this technique is whether the anatomical structure of the aMFL can be removed without biomechanical consequences for the knee joint. An answer to this can be found in the study by Palma Kries et al., presented at ISAKOS 2025, which analyzed the restriction of posterior tibial translation by the MFLs in a robotic biomechanical model. The authors revealed that the PCL is the primary stabilizer against posterior tibial translation in all tested positions of knee flexion and rotation (*P* < .05). Furthermore, they found that the aMFL and posterior MFL did not significantly restrict posterior tibial translation in neutral, internal, or external rotation (*P* > .05).^23^

## DISCUSSION

The presented technique enables anatomical positioning of the posterior root of the lateral meniscus, thereby offering favorable conditions for healing. As the meniscus is not sutured directly, but rather stabilized using an existing anatomical structure, the fixation is both secure and biologically advantageous, facilitating a more efficient healing process. By utilizing an existing anatomical structure, this method promotes faster and more efficient biological integration of the aMFL within the bone tunnel, ultimately resulting in a strong and durable reconstruction. This technique also allows for the restoration of the anatomical force vector transmitted through the aMFL to the posterior root of the lateral meniscus, as the fixation preserves the anteromedial direction of force application. Pearls and pitfalls of the technique are listed in Table 1; advantages and disadvantages are described in Table 2.

**TABLE 1:** Pearls and Pitfalls

Pearls	Pitfalls
‐ The tibial tunnel should be created in such a way that its entry point is located at the anatomical attachment site of the lateral meniscus posterior root ‐ Monitor the patient clinically and with sequential MRI examinations to assess the adequacy of healing ‐ During the healing process, use biological augmentation methods such as intra‐articular injections of PDGF	‐ This technique should preferably be used in patients with acute injuries; in chronic cases, assess whether it is possible to utilize the aMFL for fixation of the lateral meniscus posterior root

aMFL, anterior meniscofemoral ligament; MRI, magnetic resonance imaging; PDGF, platelet‐derived growth factors.

**TABLE 2:** Advantages and Disadvantages

Advantages	Disadvantages
‐ Utilization of the existing anatomical structure that enables strong, natural, and biological fixation of the defect ‐ The strong anatomical connection between the aMFL and the lateral meniscus posterior root enables robust and proper healing without the need for additional sutures within the injured posterior root	‐ It is not always possible to dissect the aMFL in a straightforward manner so as to create an effective fixation structure for the posterior root ‐ This technique may be problematic in chronic injuries, where the structure of the lateral meniscus posterior root and the aMFL may undergo remodeling that prevents proper dissection of the tissues as well as adequate healing following the application of the technique

aMFL, anterior meniscofemoral ligament.

## DISCLOSURES

The authors (R.Ś., J.M., D.K.) declare that they have no known competing financial interests or personal relationships that could have appeared to influence the work reported in this article.
